# The Analysis of Estrogen-Degrading and Functional Metabolism Genes in *Rhodococcus equi* DSSKP-R-001

**DOI:** 10.1155/2020/9369182

**Published:** 2020-08-25

**Authors:** Kejian Tian, Fanxing Meng, Qi Meng, Yan Gao, Lili Zhang, Le Wang, Yuqing Wang, Xue Li, Hongliang Huo

**Affiliations:** ^1^School of Environment, Northeast Normal University, No. 2555 Jingyue Avenue, Changchun City, Jilin Province, China; ^2^School of Life Sciences, Northeast Normal University, No. 5268, Renmin Main Street, Nanguan District, Changchun City, Jilin Province, China; ^3^Jilin Province Laboratory of Water Pollution Control and Resource Engineering, Changchun 130117, China

## Abstract

Estrogen contamination is recognized as one of the most serious environmental problems, causing widespread concern worldwide. Environmental estrogens are mainly derived from human and vertebrate excretion, drugs, and agricultural activities. The use of microorganisms is currently the most economical and effective method for biodegradation of environmental estrogens. *Rhodococcus equi* DSSKP-R-001 (R-001) has strong estrogen-degrading capabilities. Our study indicated that R-001 can use different types of estrogen as its sole carbon source for growth and metabolism, with final degradation rates above 90%. Transcriptome analysis showed that 720 (E1), 983 (E2), and 845 (EE2) genes were significantly upregulated in the estrogen-treated group compared with the control group, and 270 differentially expressed genes (DEGs) were upregulated across all treatment groups. These DEGs included ABC transporters; estrogen-degrading genes, including those that perform initial oxidation and dehydrogenation reactions and those that further degrade the resulting substrates into small molecules; and metabolism genes that complete the intracellular transformation and utilization of estrogen metabolites through biological processes such as amino acid metabolism, lipid metabolism, carbohydrate metabolism, and the tricarboxylic acid cycle. In summary, the biodegradation of estrogens is coordinated by a metabolic network of estrogen-degrading enzymes, transporters, metabolic enzymes, and other coenzymes. In this study, the metabolic mechanisms by which *Rhodococcus equi* R-001 degrades various estrogens were analyzed for the first time. A new pollutant metabolism system is outlined, providing a starting point for the construction of engineered estrogen-degrading bacteria.

## 1. Introduction

Environmental estrogen is a type of endocrine disruptor (EDC) that has generated significant worldwide concern. After entering an organism, it interferes with hormone synthesis, secretion, transport, binding, reactions, and metabolism in ways that affect normal homeostasis and endanger reproduction, development, and behavior [1]. Estrogen is a fat-soluble substance whose basic nucleus is a steroid ring formed by the condensation of three six-membered rings and one five-membered ring [2]. Common estrogens include E1, E2, and EE2, which differ in the functional groups at C-17 (Figure S1). The estrogenic activity of 17*α*-ethynyl estradiol (EE2) is about 2.5 times greater than that of 17*β*-estradiol (E2), and the estrogenic activity of estrone (E1) is much lower (about 2.54% of E2) than that of E2. At present, estrogens in the environment are mainly derived from human and animal excreta [3] or enter the environment directly through composting. The large-scale use of estrogen drugs in the aquaculture industry has aggravated this process. In addition, sewage treatment plants do not completely remove estrogen, and it can be discharged into natural water bodies through effluent. Estrogens accumulate easily in organisms after entering the environment; their endocrine disruption is therefore stronger than that of other EDCs, and their impact on the environment is particularly significant [4–6]. Studies have shown that very low concentrations of estrogen can cause feminization of males in aquatic organisms [7, 8]. Estrogen can also affect the human body through direct contact with the skin and via enriched food chains. It can cause endocrine system disorders, female tumors, cancer, male infertility, and precocious puberty. In addition, it may interfere with gender differentiation in the brain, accelerate growth and estrus, increase weight, and cause immune disorders. Therefore, the World Health Organization has classified estrogens as group 1 carcinogens [9] (https://monographs.iarc.fr/list-of-classifications).

The microbial degradation of estrogen is mainly accomplished by bacteria and a small number of fungi and algae. Most estrogen-degrading bacteria have been identified from activated sludge, farmland soil, or compost in sewage treatment plants. At present, more than one hundred estrogen-degrading microorganisms have been reported [10–34], and the estrogen degradation efficiency of some strains can reach more than 90% within a specific time period (Table S1). For example, *Actinomycetes* have very good substrate adaptation to high concentrations of estrogen and exhibit no delay in cell growth. At the same time, their estrogen degradation efficiency and degradation rate are relatively high. Among the 16 *Actinomycetes* that have been screened, five strains of high-efficiency estrogen-degrading bacteria have been identified, all belonging to the genus *Rhodococcus*. sp. Their degradation rates for estrogens E1, E2, E3, and EE2 are all greater than 95%. For example, *Rhodococcus equi* Y50156 and *Rhodococcus zopfii* Y50158 have a degradation rate > 95% at 24 h for 100 mg/L concentrations of the four estrogens listed above [11], and *Rhodococcus rubber* KC4 has a 99% degradation rate at 24 h for E2a at a concentration of 3 mg/L [15]. In our previous study, the degradation rate at 72 h for 50 mg/L E2 by *R. equi* DSSKP-R-001 was 97% [35], and the degradation rates at 96 h of 30 mg/L E1, E2, and EE2 were 100%, 100%, and 90%, respectively. Therefore, the *Rhodococcus*. sp shows significant promise for construction of engineered, estrogen-degrading bacteria.

Microbial degradation of estrogen is mainly accomplished through enzymatic reactions, and the genes encoding these enzymes are key to the biodegradation of organic matter. The genes *OecA*, encoding 3*β*, 17*β*-hydroxysteroid dehydrogenase; *OecB*, encoding estrone-4-hydroxylase; and *OecC*, encoding 4-hydroxyestrone 4,5-dioxygenase, are the key estrogen degradation genes in *Sphingomonas* sp. KC8 [36]. When *Acinetobacter* sp. DSSKY-A-001 was cultured with estradiol as the sole carbon source, Huo found that the dioxygenase gene, *RspwpGM002188*; the catechol 1,2 dioxygenase gene, *RspwpGM001668*; and the 7*α*-hydroxysteroid dehydrogenase gene, *RspwpGM001333*, were involved in different E2 degradation stages and had high expression levels [37]. Ye et al. [38] used plasmid construction and heterologous expression to demonstrate that *HSD*, which encodes a 17*β*-hydroxysteroid dehydrogenase in *Rhodococcus* sp. P14, was related to the bacterium's E2 degradation ability. In 2009, Petrusma et al. [39] successfully expressed the *kshA* and *kshB* genes from *Rhodococcus rhodochrous* DSM 43269 in *E. coli* and demonstrated that *kshA* catalyzed substrate hydroxylation due to the reductase properties of *kshB*. In 2011, Petrusma et al. [40] analyzed the enzymatic properties of five *kshA* gene products from *Rhodococcus rhodochrous* DSM43269. The five homologs, *kshA1* to *kshA5*, belonged to four different gene clusters and exhibited significant overlap in their steroid substrates. Although the catabolic activity of *kshA1* was specific to cholic acid, *KshA5* was able to grow on many classes of steroids with no obvious substrate preference.

Although many detailed estrogen degradation pathways have been proposed [9, 27, 41], studies on the intracellular metabolism of estrogen have not been reported. At the same time, estrogen pollution is currently caused by multiple channels and multiple estrogens, and the study of bacterial estrogen degradation therefore has great practical significance. The purpose of this study was to characterize (1) the ability of *R. equi* DSSKP-R-001 to degrade different types of estrogen, (2) the effects of different types of estrogen on gene expression in R-001, and (3) the mechanism of estrogen metabolism in bacterial cells. We performed experiments with single and mixed carbon sources and used transcriptome sequencing to study gene expression in response to different estrogen treatments and explore the mechanism of estrogen metabolism. This paper reports the first attempt to study gene expression of *R. equi* in various estrogen cultures using transcriptomics. It further clarifies the metabolic response of *R. equi* to estrogens and provides guidance for the construction of functional strains.

## 2. Materials and Methods

### 2.1. Strain and Chemicals

The R-001 strain used in this study was isolated from soil near a long-term estrogen-contaminated contraceptive plant in Beijing and was preserved at the China General Microbiological Culture Collection Center. Thallus was cultured in LB medium and placed in a constant-temperature shaking incubator at 30°C for 120 h at 120 rpm. The OD600 of the bacterial liquid was measured using a microplate reader (BioTek-ELX 800). A bacterial suspension of OD600 = 1 was used for degradation experiments. Studies have shown that estrogen concentration does not affect degradation mechanism: the degradation mechanisms of strains grown with high concentrations of estrogen are the same as those used when estrogen is present in typical water concentrations [42]. Therefore, the bacterial suspension was added to mineral basal medium supplemented with 30 mg/L estrogen or glucose at an inoculum rate of 3% by volume.

Estrone (E1), 17*β*-estradiol (E2), and 17*α*-ethynyl estradiol (EE2) used in this study were all 99% pure standard materials produced by Aladdin; acetonitrile in the HPLC mobile phase was produced by Sigma.

### 2.2. Estrogen Concentrations and Detection of Intermediate Products

A high-performance liquid chromatograph (HPLC; Shimadzu Co.) was used with a Zorbax Eclipse Plus C18 column (150 × 4.6 mm, 3.5 mm). Injected materials were detected at room temperature using a flow rate of 0.8 mL/min and a wavelength of 220 nm with an injection volume of 10 *μ*L and a flow ratio of 1 : 1. The retention times of E1, E2, and EE2 were 10.853 min, 8.928 min, and 12.375 min, respectively. The standard curve (*R*^2^ > 0.999) was prepared with OriginPro 2017 software based on the concentration and peak area of each substance, and sample estrogen concentrations were calculated from the curve. An Applied Biosystems PE Sciex API 2000 MDS LC/MS/MS System was used to analyze intermediate products in the process of estrogen degradation. The setting conditions were the same as those described for the HPLC measurements. At a temperature of 0°C, negative ions were used to scan in the range of 50–600 Da, and the collision energy (CE) was set to 35 ev.

### 2.3. RNA Extraction, Library Construction, and Transcriptome Sequencing

Bacteria were cultured as above, using estrogen for the treatment groups and glucose for the control group. Total RNA was extracted using the TRIzol reagent (Life Technologies, CA, USA). RNA quality control was performed in several steps:

1. RNA degradation and potential contamination were monitored on 1% agarose gels
2. RNA purity (OD260/OD280, OD260/OD230) was assessed using a NanoPhotometer® spectrophotometer (Implen, CA, USA).
3. RNA integrity was measured using a Bioanalyzer 2100 (Agilent, Santa Clara, CA).

Briefly, the rRNA was removed from 1 microgram of total RNA using the Ribo-Zero Magnetic Gold Kit (Epicentre Biotechnologies, Madison, WI, USA). The TruSeq RNA Sample Prep Kit v2 (Illumina, San Diego, CA, USA) was used for library construction. RNA was fragmented into small pieces using the Elute Prime Fragment Mix. First-strand cDNA was synthesized with First-Strand Master Mix and SuperScript II (Invitrogen, Carlsbad, CA, USA) reverse transcription (25°C for 10 min; 42°C for 50 min; 70°C for 15 min). After product purification with Agencourt RNAClean XP Beads (Beckman Coulter, CA, USA), the second-strand cDNA library was synthesized using Second Strand Master Mix and a dATP, dGTP, dCTP, and dUTP mix (1 h at 16°C). Purified fragmented cDNA was end-repaired (30 min at 30°C) and purified with AMPure XP Beads (Beckman Coulter, CA, USA). Addition of the poly(A) tail was performed with A-tailing Mix (30 min at 37°C) prior to the ligation of sequencing adapters (10 min at 30°C). The second-strand cDNA was degraded using the Uracil-N-Glycosylase enzyme (10 min at 37°C), and the product was purified using AMPure XP Beads. Several rounds of PCR amplification with PCR Primer Cocktail were performed to enrich the cDNA fragments, and the PCR products were purified using AMPure XP Beads.

Clustering of the barcoded samples was performed on a cBot Cluster Generation System according to the manufacturer's instructions. After cluster generation, sequencing was performed using the Illumina HiSeq™ 2500 platform with paired-end 150 bp reads. Raw reads were submitted to the NCBI Sequence Read Archive under the accession number PRJNA564375.

### 2.4. Read Filtering and Alignment

Raw data were filtered by (1) removing reads with ≥10% unidentified nucleotides (N), (2) removing reads in which >50% of the bases had PHRED quality scores of ≤20, and (3) removing reads that aligned to the barcode adapter using FASTP (https://github.com/OpenGene/fastp). Clean reads were mapped to the reference genome using Bowtie2 [43] (version 2.2.8) allowing no mismatches, and reads that mapped to ribosomal RNA were removed. Retained reads were aligned to the reference genome using Bowtie2 [43] (version 2.2.8) to identify known genes, and gene expression levels were quantified using RSEM [44].

### 2.5. Analysis of Differentially Expressed Genes

Gene expression data were normalized using the fragments per kilobase of transcript per million mapped reads (FPKM) method to eliminate the influence of different gene lengths and sequencing depths on the calculation of gene expression. The edgeR package (http://www.r-project.org/) was used to identify differentially expressed genes (DEGs) with fold changes ≥ 2 and a false discovery rate-adjusted *P* value (*q* value) of <0.05. DEGs were then subjected to Gene Ontology (GO) and Kyoto Encyclopedia of Genes and Genomes (KEGG) pathway enrichment analyses, again using a *q* value threshold of 0.05.

### 2.6. Real-Time Quantitative PCR Validation

Extracted RNA was subjected to reverse transcription using the HiScript 1st Strand cDNA Synthesis Kit (Jizhen Biology, China). The qPCR reaction was carried out on a CFX96 instrument (Bio-Rad, USA) using AceQ qPCR SYBR Green Master Mix (Jizhen Biology, China). The reaction measurements were performed in biological triplicate. The reaction system consisted of 5 *μ*L 2x SYBR Green Mix, 1 *μ*L Primer_F+R (each 10 *μ*M), 2 *μ*L cDNA, and 3 *μ*L ddH_2_O. Reaction conditions and primer designs are presented in Tables S2 and S3. The results were expressed relative to the expression levels of the recA reference gene in each sample using the 2^−*ΔΔ*Ct^ method.

## 3. Results and Discussion

### 3.1. Degradation of Estrogen

**Figure 1:**
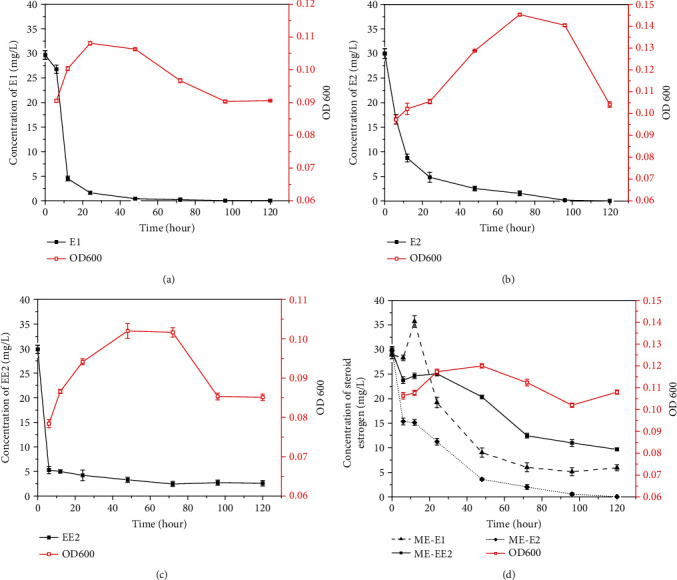
Biodegradation curve and growth curve of strain R-001 on three estrogens. (a) R-001 degrading E1. (b) R-001 degrading E2. (c) R-001 degrading EE2. (d) R-001 degrading mixed estrogens.

The degradation of different estrogens and the growth of R-001 were measured by high-performance liquid chromatography and a microplate reader. During the biodegradation of E1, the strain entered the logarithmic growth phase within 24 h. The E1 degradation rate reached 83.3% at 12 h and 98.6% at 48 h. The strain then gradually entered the decline phase with the depletion of its carbon source (Figure 1(a)). The biodegradation of E2 by R-001 was different from the biodegradation of E1. The substrate degradation rate was slower, and the strain showed an obvious lag phase. At 48 h, the strain entered the logarithmic growth phase, at which time the degradation rate reached 91.7%. After 96 h, E2 was completely degraded, and the strain entered the decline phase (Figure 1(b)). Similar to its biodegradation of E1, R-001 rapidly degraded EE2, and the degradation rate reached 84.7% at 24 h. The strain entered the logarithmic growth phase, and the degradation rate reached 90% at 72 h before entering the decline phase (Figure 1(c)). These experiments showed that R-001 can use different types of estrogen as a sole carbon source and that it has good biodegradation abilities for estrogens, with final degradation rates above 90%.

Nonetheless, there are many different estrogen contaminants in natural waters, and it is therefore particularly important to study the growth of estrogen-degrading strains and their estrogen-degrading ability in the presence of multiple estrogen types. The natural estrogens E1 and E2 and the artificial estrogen EE2 were selected as target pollutants for the culture system. E1 concentration increased slightly at 12 h and rapidly decreased after 12 h. This may be due to rapid degradation of E2 and EE2 at the beginning of the experiment, resulting in an accumulation of E1 as an intermediate product for a short time. The degradation trends of E2 and EE2 were similar. Biodegradation was rapid before 12 h and slowed after 12 h. The degradation rate of EE2 was 58.7% at 72 h and that of E2 was 97.3%. at 97 h. After that, the degradation process remained essentially stagnant (Figure 1(d)). During exposure to high concentrations of mixed estrogen pollutants, the estrogen-degrading bacteria R-001 could therefore use different types of estrogen for growth and metabolism, including both natural and artificial estrogens. Compared with their behavior under single estrogen culture conditions, the estrogen-degrading strains had slower rates of pollutant removal in the mixed substrate culture, and the final residual pollutant concentrations were higher. This may be related to the estrogen type, the high estrogen concentrations, and the relatively brief experimental period. Because the strain did not completely enter the decay phase at the end of the experiment, we can speculate that an extension of the experimental cycle may result in further removal of the mixed estrogens.

### 3.2. Overview of Transcriptome Sequencing Results

#### 3.2.1. Data Reliability Analysis

We used transcriptome sequencing to study the key genes that play a role in R-001's degradation of estrogen. Summary data from the sequencing experiment are shown in Table 1. After removing low-quality data and rRNA, a total of 40,916,088 reads were obtained for the assembly and analysis. Greater than 80% of the clean reads mapped to the reference genome, suggesting that the transcriptome sequence data were accurate and reliable. Sequence saturation analysis showed that as the amount of sequence data increased, the rate of detected transcripts plateaued, indicating that the number of detected transcripts had become saturated (Figure S2).

To validate the transcriptome sequencing results, the expression of nine selected DEGs (*choD*, *ksdI*, *hsaC*, *hsaD*, *kshA*, *tesI*, *HSD17B4*, *ksdD*, and *hsdA*) was measured by real-time quantitative PCR in response to estradiol treatment (Figure S3). Their expression was consistent with that documented in the RNA-Seq experiment, with the exception of *hsaC*, whose expression was significantly higher.

#### 3.2.2. DEGs under Different Estrogen Treatments

**Figure 2:**
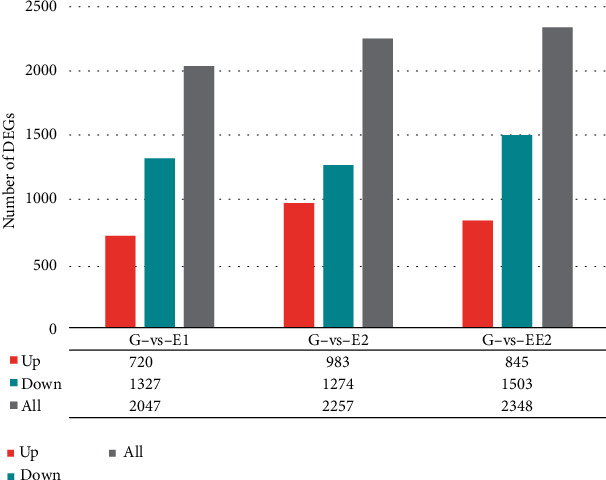
General overview of differentially expressed genes in R-001 treated with estrogens.

**Figure 3:**
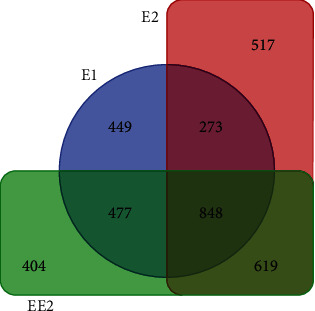
Venn diagram of differentially expressed genes in different estrogen treatment groups.

Compared with the glucose control group (Figure 2), R-001 treated with estrogen had 2047 DEGs (720 up and 1327 down), 2257 DEGs (983 up and 1274 down), and 2348 DEGs (845 up and 1503 down) when grown with E1, E2, and EE2, respectively (FDR < 0.05, ∣log_2_FC | >1). There were 848 DEGs common to all treatment groups (578 down and 270 up), and 449, 517, and 404 DEGs unique to each treatment group (Figure 3).

#### 3.2.3. Functional Classification of DEGs

Estrogens have very similar chemical structures. E1, E2, and EE2 were therefore selected as representative estrogens in experiments designed to (1) identify DEGs associated with estrogen biodegradation and metabolism and (2) investigate the mechanisms of estrogen metabolism.

**Figure 4:**
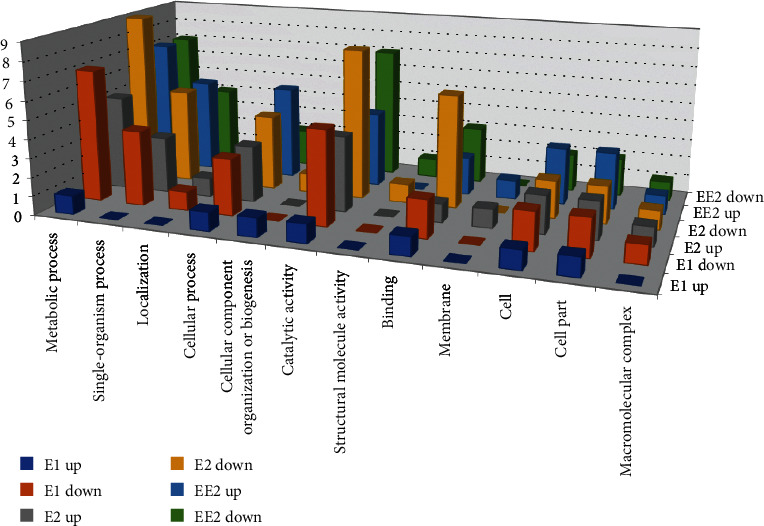
GO functional enrichment histogram of strain R-001 under different estrogen conditions.

GO analysis assigns putative functional annotations to DEGs and identifies significantly enriched GO terms in the DEG set, thereby predicting the main biological functions of the DEGs. According to the annotation results (Figure 4, Table S4), 12, 19 and 18 DEGs from the E1, E2, and EE2 treatments received GO annotations, respectively. Among the annotated DEGs, 36 were annotated with the term metabolic process, 21 with single-organism process, and 18 with cellular process in the Biological Process GO category. Likewise, 29 genes were annotated with the term catalytic activity and 15 with the term binding in the Molecular Function GO category. Twelve DEGs were annotated with the term cell and 12 with the term cell part in the Cellular Component GO category. The results are consistent with DEGs functioning in metabolism and in the catalytic activity of enzymes. Five of the common DEGs (C7H75_RS14070, C7H75_RS03905, C7H75_RS14550, C7H75_RS18270, and C7H75_RS18640) received GO annotations. C7H75_RS18270 was upregulated 7.13-fold and was annotated as a chaperone protein (Chaperonin GroES, groS). Studies have shown that molecular chaperones are part of the protein folding mechanism and help to maintain cellular homeostasis [45, 46]. GroEL/GroES overexpression can counteract nascent protein misfolding, increase adaptability, and expand “mutation space”. It can also increase bacterial resistance or tolerance, minimizing drug damage and promoting survival and growth in exponential culture [47, 48].

KEGG functional annotation results indicated (Figure S4) that DEGs were enriched in ABC transporters; amino sugar and nucleotide sugar metabolism; aminobenzoate degradation; arginine biosynthesis; base excision repair; benzoate degradation; *β*-alanine metabolism; biosynthesis of unsaturated fatty acids; biotin metabolism; butyric acid metabolism; caprolactam degradation; chlorocyclohexane and chlorobenzene degradation; degradation of aromatic compounds; gene replication; fatty acid biosynthesis; fatty acid degradation and metabolism; fluorobenzoate degradation; lysine degradation; mismatch repair; niacin and nicotinamide metabolism; phenylalanine metabolism; polyketide biosynthesis; styrene degradation; thiamine metabolism; toluene degradation; tyrosine metabolism; and proline, leucine, and isoleucine degradation. The biodegradation of estrogens and other functions of significant DEGs will be discussed in greater detail below.

### 3.3. DEGs Related to Estrogen Degradation

HSD plays a major role in the dehydrogenation of steroid hormones. Both 17*β*-HSD recombinant strains and purified proteins can efficiently convert E2 into E1 by C17 dehydrogenation [49], and a similar role for HSD has been reported in some fungi and *Pseudomonas* sp. [50–52]. In this study, significantly upregulated (13.60-fold and 13.41-fold) in response to E2 and EE2 treatments (Table 2), suggesting that *17β-HSD* may be involved in C17 dehydrogenation during the degradation of steroid estrogens. E1 production was detected during E2 degradation (Figure S5), indicating that E2 may be degraded to E1 by *17β-HSD* during the initial stages of the degradation process. The accumulation of E1 during the first 12 h of biodegradation further confirmed this view (Figure 1(d)).

**Figure 5:**
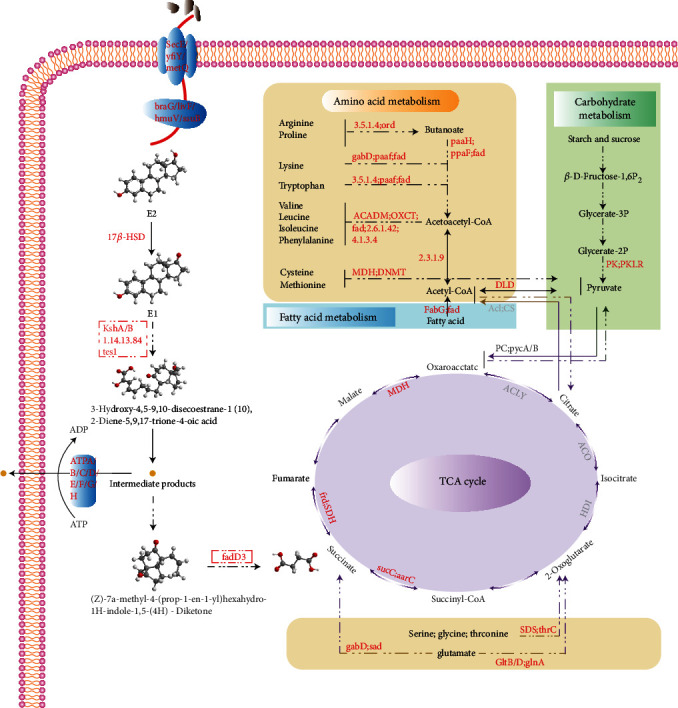
A diagram of the metabolic mechanisms of DEGs that were significantly upregulated in Strain R-001 under different estrogen treatments. Upregulated genes are shown in red font, and genes whose expression did not change are shown in gray. Refer to the text for additional details. The gene in the red-dashed line indicates that we believe that it plays a role in the corresponding degradation process, but the specific reaction process involved in it has not been identified.

3-anthrone-9*α*-hydroxylase (KSH) is a two-component iron-sulfur monooxygenase that is widely distributed among sterol-degrading bacteria [39, 53–55]. It consists of a terminal oxidase (kshA) and a reductase (kshB) [56]. KSH can add a hydroxyl group (9*α*-OH) at the C9 position of the polycyclic ring and participates with KSTD in carbon skeleton cleavage of the steroid nucleus B ring [57, 58]. Transcriptome results indicated that *kshA* (C7H75_RS02785/C7H75_RS08645) encoded a 3-indolone-9*α*-hydroxylase oxidase subunit that was upregulated 14-fold, 12.4/11.71-fold, and 12.8-fold in the E1, E2, and EE2 treatment groups (Table 2), respectively. The *kshB* gene (C7H75_RS18910) encoded a 3-indolone-9*α*-hydroxylase reductase subunit and was upregulated 1.03-fold in the EE2 group only. By contrast, *kstd* [59–62], whose protein functions with KSH in B ring cleavage, was not identified in the E1 group and was downregulated in both the E2 and EE2 groups. The 3-oxo-5*α*-steroid 4-dehydrogenase gene *tesI* (C7H75_RS04150) was upregulated 14.1-fold, 13.57-fold, and 12.31-fold in the E1, E2, and EE2 groups, respcetively. Previously, Horinouchi et al. [63, 64] demonstrated that *tesI* is involved in the biodegradation of androsterone. It induces expression of testosterone degradation genes in *Comamonas testosteroni* TA441, indicating that its protein not only degrades testosterone but also functions in the degradation of other steroid substances. In addition, a gene encoding 4-hydroxyacetophenone monooxygenase was downregulated in the E2 group but upregulated by 1.16-fold in the E2 24 h group (data not shown), suggesting that it may function in the initial step of E2 degradation. The above genes may play a role in the degradation of E1 to 3-hydroxy-4,5-9,10-disecoestrane-1 (10), 2-diene-5,9,17-trione-4-oic acid (Figure 5, Figure S5), but their specific roles require further study. Finally, *ksdI* (C7H75_RS24110), *fadD3* (C7H75_RS03705), *hsaC* (C7H75_RS25395), *hsaD* (C7H75_RS00645), and *hsaA* (C7H75_RS02355) were also significantly upregulated in the estrogen treatment groups.

### 3.4. DEGs Related to Metabolism

Metabolic processes provide the necessary energy for growth and organic matter degradation and play an important role in biodegradation. The DEGs shown in Table S5 are simultaneously identified in at least two treatment conditions. Their mechanisms of action are shown in Figure 5.

#### 3.4.1. Transport

ABC transporters are involved in the transport of biological substances across cell membranes. They use the energy of ATP hydrolysis to transport a variety of substrates, including inorganic ions, sugars, amino acids, drugs, and toxic intermediates [65]. They play particularly important roles in cell nutrient uptake and toxic metabolite efflux [66, 67]. ABC transporter genes were significantly upregulated in response to estradiol treatment (Table 3, Figure 5). Although other transporters such as AtpA (ATP synthase A chain) and secE (preprotein translocase subunit) also play a role in estrogen transport, they showed little differential expression, and secE expression was significantly upregulated only under 50 mg/L estradiol treatment (data not shown). In addition, membrane ion transporters can promote substrate uptake by generating electrochemical gradients. However, ion transport DEGs in our experiment were downregulated, further suggesting an important role for ABC transporters in substrate uptake, although their specific mechanism of action will require further study.

#### 3.4.2. Amino Acid Metabolism

DEGs were involved in 10 amino acid metabolic pathways (Table S5), described briefly in the following paragraphs. Proline, leucine, and isoleucine are metabolized to produce propionyl-CoA, acetoacetyl-CoA, acetyl-CoA, methacryloyl-CoA, and succinyl-CoA to generate NADH/FADH_2_ for electron transport and oxidative phosphorylation. Tyrosine is converted to fumarate and acetoacetate, and phenylalanine is converted to fumarate, succinate, and acetyl-CoA. Tryptophan is first converted to acetoacetyl-CoA, then to acetyl-CoA. Cysteine is oxidized to cysteine sulfuric acid, which is then converted to pyruvic acid by transamination. All of the above metabolites eventually enter the TCA cycle for further oxidation and energy production. The metabolism of glycine, serine, and threonine is a dehydratase-catalyzed deamination reaction that converts glycine and serine to pyruvate. Threonine is metabolized to homoserine or *α*-ketobutyric acid and then enters the cysteine/methionine metabolic pathway and the proline/leucine/isoleucine biosynthetic pathway or the propionic acid metabolic pathway to produce ATP (Figure 5).

Lysine is primarily metabolized by a transamination reaction, converted first to glutaryl coenzyme A and acetoacetyl-CoA and finally to acetyl-CoA. Histidine can eventually be metabolized to form glutamate, which is then converted to *α*-ketoglutarate by deamination via glutamate dehydrogenase. Metabolites of lysine and histidine enter the TCA cycle to produce energy for cell growth and development (Figure 5).

The metabolism of alanine produces pyruvic acid by transamination, which then enters the glycolysis/gluconeogenesis pathway. The aspartate metabolic pathway involves dehydrogenation to produce oxaloacetate or transamination to produce fumaric acid. Glutamate is converted to glutamine, which can be converted to proline and arginine by transamination or to *α*-ketoglutarate by glutamate dehydrogenase, after which it enters the TCA cycle [68]. In addition, glutamate can be transformed into succinic semialdehyde and then to succinic acid for the TCA cycle (Figure 5). Upregulation of amino acid metabolism genes promotes the production of intermediates for other metabolic processes such as carbohydrate and lipid metabolism and contributes to the energy production required for cellular processes. This is similar to the process by which Tween80 promotes the biodegradation of phenanthrene by *Sphingomonas* sp. GY2B and the biodegradation of nicotine by *Aspergillus oryzae* 112822 [68, 69].

#### 3.4.3. Carbohydrate Metabolism

Carbohydrate metabolism provides the energy necessary for bacterial growth and substrate uptake. Glycolysis/gluconeogenesis supplies energy and maintains sugar levels in cells. After metabolism of starch and sucrose, glucose enters glycolysis to be converted to pyruvic acid, which is further metabolized to acetyl-CoA for use in the TCA cycle to generate energy. The DEG *deoC* of the parallel pentose phosphate metabolic pathway encodes deoxyribose-phosphate aldolase and was upregulated 13.45-fold and 12.31-fold in the E2 and EE2 treatment groups, respectively (Table S5, Figure 5). In addition to acting as a biocatalyst, this enzyme plays an important role in the pentose phosphate pathway, which prevents oxidative stress and maintains cellular homeostasis [70].

#### 3.4.4. Fatty Acid Metabolism

Fatty acid metabolism can produce acetyl-CoA, which eventually enters the TCA cycle to provide the energy needed for cellular processes and substrate degradation (Figure 5). Liu et al. [68] showed that upregulation of fatty acid metabolism genes is an important complementary mechanism that promotes the biodegradation of phenanthrene. In our study, 19 genes associated with fatty acid metabolism were differentially expressed in estrogen-treated groups. For example, *fabG* was upregulated more than 10-fold in multiple treatment groups (Table S5). FabG belongs to the short-chain dehydrogenase reductase (SDR) family [71]; it is ubiquitous in bacteria and plays an important role in the synthesis of type II fatty acids [72] and fatty acid metabolism [73].

#### 3.4.5. Tricarboxylic Acid Cycle

Pyruvic acid is an important link between amino acid, carbohydrate, and lipid metabolism and the TCA cycle. Eight significantly upregulated DEGs were associated with pyruvate metabolism (Table S5). Pyruvate metabolites such as oxaloacetic acid and other intermediates enter the TCA cycle to produce energy.

The TCA cycle is the final destination for products of carbohydrate, lipid, and amino acid metabolism; they are oxidized via the TCA cycle to produce reducing equivalents (NADH, FADH_2_, and GDP) for oxidative phosphorylation and, ultimately, ATP production. The TCA cycle also produces intermediates required for the biosynthesis of cellular components (Figure 5). A large number of DEGs were associated with the TCA cycle (Table S5).

The removal of estrogen organic pollutants by R-001 occurs mainly through biometabolism, which converts the pollutants into metabolites and energy required for cell growth. This process is complex and reversible. Organic materials can provide intermediates for the TCA cycle through fatty acid, carbohydrate, and amino acid metabolism. Conversely, the TCA cycle can provide precursors for cell components and energy for cellular processes. Oxidative phosphorylation can use reduced energy from the TCA cycle to produce ATP. The above biological processes are coordinated with one another to provide energy and materials for bacterial growth and reproduction, as well as the degradation of organic matter.

## 4. Conclusion

In this paper, the estrogen-degrading bacterium *R. equi* DSSKP-R-001 was shown to degrade estrogens with high efficiency, particularly estrone and 17*β*-estradiol. Its rate of estrogen removal was somewhat reduced in the presence of mixed estrogens. For the first time, we used high-efficiency, estrogen-degrading bacteria to investigate mechanisms of estrogen degradation; previous reports have used only a single type of estrogen. Our study shows that in addition to estrogen-degrading enzymes, transporters and metabolism-related enzymes also play an important role in converting macromolecular organics into the energy and materials needed for growth.

## Figures and Tables

**Table 1:** Transcriptome sequence data after filtering and mapping to the reference genome.

Sample name	Raw reads	Clean reads (%)	Total reads (rRNA removed)	Percent mapped reads (%)
G	195,310,926	194,813,492 (99.75%)	10,537,190	82.90
E1	115,668,214	115,284,564 (99.67%)	9,237,730	85.82
E2	43,752,690	43,637,284 (99.74%)	9,873,728	88.88
EE2	43,013,658	42,863,230 (99.65%)	11,267,440	98.29

**Table 2:** DEGs related to estrogen degradation by R-001under different estrogen treatment conditions.

Gene ID	Gene name	Description	Log_2_(FC)		
			G vs. E1	G vs. E2	G vs. EE2
C7H75_RS21980	*17β-HSD*	17beta-estradiol 17-dehydrogenase	—^a^	13.60	13.41
C7H75_RS02785	*kshA*	3-ketosteroid-9-alpha-hydroxylase oxygenase subunit	14	12.4	12.8
C7H75_RS08645	*kshA*	3-ketosteroid-9-alpha-hydroxylase oxygenase subunit	—	11.71	—
C7H75_RS18910	*kshB*	3-ketosteroid-9-alpha-hydroxylase reductase subunit	—	—	1.03
C7H75_RS04150	*tesI*	3-oxo-5alpha-steroid 4-dehydrogenase	—	10.91	11.95
C7H75_RS24110	*ksdI*	Steroid Delta-isomerase	—	14.33	14.42
C7H75_RS03705	*fadD3*	AMP-binding enzyme	2.76	—	1.89
C7H75_RS25395	*hsaC*	Glyoxalase	—	6.02	12.88
C7H75_RS00645	*hsaD*	Alpha/beta hydrolase	—	11.83	—
C7H75_RS02355	*hsdA*	Short-chain dehydrogenase/reductase	—	13.11	12.36

^a^No significant differential expression in this treatment group.

**Table 3:** DEGs related to estrogen transport in R-001 under different estrogen treatments.

Gene ID	Gene name	Description	Log2(FC)		
			G vs. E1	G vs. E2	G vs. EE2
C7H75_RS20820	*braF*	Branched-chain amino acid ABC transporter substrate-binding protein	12.59	—^a^	11.33
C7H75_RS19315	*braG*	ABC transporter ATP-binding protein	15.72	12.89	15.06
C7H75_RS20815	*livF*	ABC transporter ATP-binding protein	13.56	—	12.77
C7H75_RS12365	*hmuV*	Heme ABC transporter ATP-binding protein	14.05	11.95	12.5
C7H75_RS12945	*fepD*	ABC transporter transmembrane protein	2.1	—	1.61
C7H75_RS02430	*yfiY*	Substrate-binding lipoprotein	1.56	—	1.84
C7H75_RS04265		ABC transporter substrate-binding protein	12.68	—	12.3
C7H75_RS11220		Iron ABC transporter substrate-binding protein	1.73	—	1.71
C7H75_RS21070	*ssuB*	Nitrate ABC transporter ATP-binding protein	—	12	14.78
C7H75_RS08765		ABC transporter substrate-binding protein	15.86	12.91	14.19
C7H75_RS22280		Lipoprotein	3.33	—	4.27
C7H75_RS02015		ABC transporter	—	11.88	11.6
C7H75_RS05615		Mce family protein mce3	1.06	—	2.45
C7H75_RS10320	*metQ*	ABC transporter substrate-binding protein	—	1.42	2.27

^a^No significant differential expression in this treatment group.

## Data Availability

The data used to support the findings of this study are included within the article.

## Supplementary Materials

Supplementary 1 Supplementary figures: Figure S1: estrogen chemical structural formula. Figure S2: sequencing saturation map of strain R-001. a: E1, b: E2, c:EE2, d: G. Figure S3: differential expression of genes under estradiol treatment, comparison between RT-qPCR and RNA-seq. Figure S4: enriched bubble chart of KEGG for R-001 differentially expressed gene under steroid estrogen treatment. A: G-vs-E1, B: G-vs-E2, C: G-vs-EE2. Rich Factor refers to the ratio of the number of DEGs to the total number of genes in the pathway. A larger Rich Factor indicates a higher degree of enrichment. The closer the *q* value is to zero, the more significant the enrichment is. Figure S5: estradiol metabolic pathway map of strain R-001 under E2 treatment. Red border: enzymes encoded by upregulated genes, green border: enzymes encoded by downregulated genes, and red+green border: enzymes encoded by both upregulated and downregulated genes.

Supplementary 2 Supplementary tables: Table S1: isolated estrogen-degrading bacteria. Table S2: the primer information in this study. Table S3: qPCR reaction procedure. Table S4: GO function enrichment analysis result. Table S5: DEGs related to metabolism of strain R-001 under different estrogen treatment conditions.
