# Supplementary material for: The Analysis of Estrogen-Degrading and Functional Metabolism Genes in Rhodococcus equi DSSKP-R-001
Source: Int J Genomics. 2020 Aug 25;2020:9369182. doi: 10.1155/2020/9369182 (PMC7471831; doi:10.1155/2020/9369182)
Supplement: Supplementary 1 — Supplementary figures: Figure S1: estrogen chemical structural formula. Figure S2: sequencing saturation map of strain R-001. a: E1, b: E2, c:EE2, d: G. Figure S3: differential expression of genes under estradiol treatment, comparison between RT-qPCR and RNA-seq. Figure S4: enriched bubble chart of KEGG for R-001 differentially expressed gene under steroid estrogen treatment. A: G-vs-E1, B: G-vs-E2, C: G-vs-EE2. Rich Factor refers to the ratio of the number of DEGs to the total number of genes in the pathway. A larger Rich Factor indicates a higher degree of enrichment. The closer the q value is to zero, the more significant the enrichment is. Figure S5: estradiol metabolic pathway map of strain R-001 under E2 treatment. Red border: enzymes encoded by upregulated genes, green border: enzymes encoded by downregulated genes, and red+green border: enzymes encoded by both upregulated and downregulated genes. [file 9369182.f1.doc]

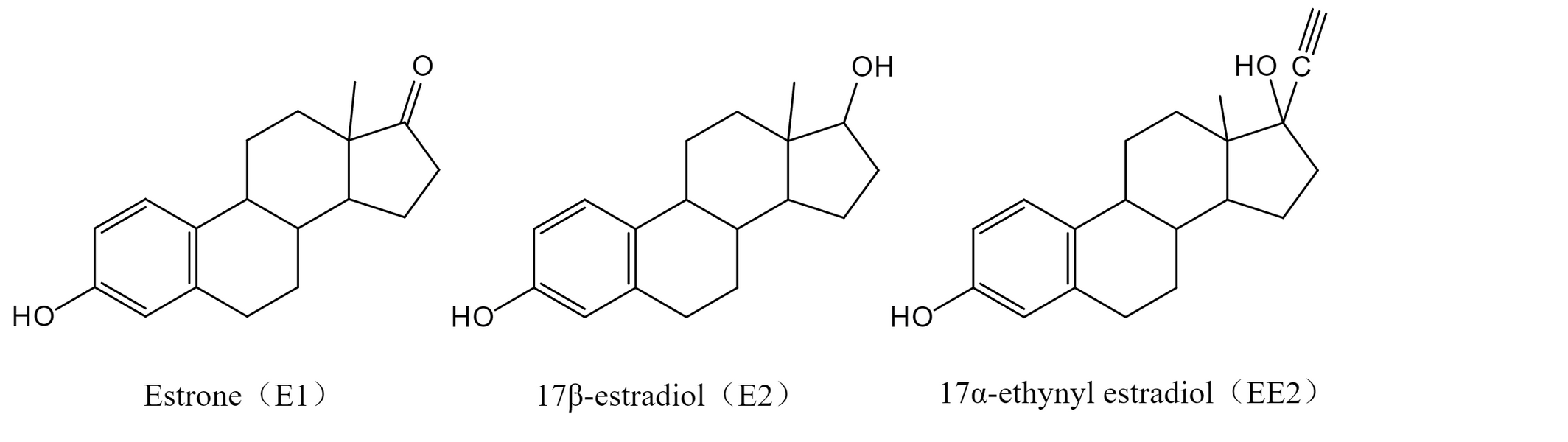


**Fig. S1** Estrogen chemical structural formula.


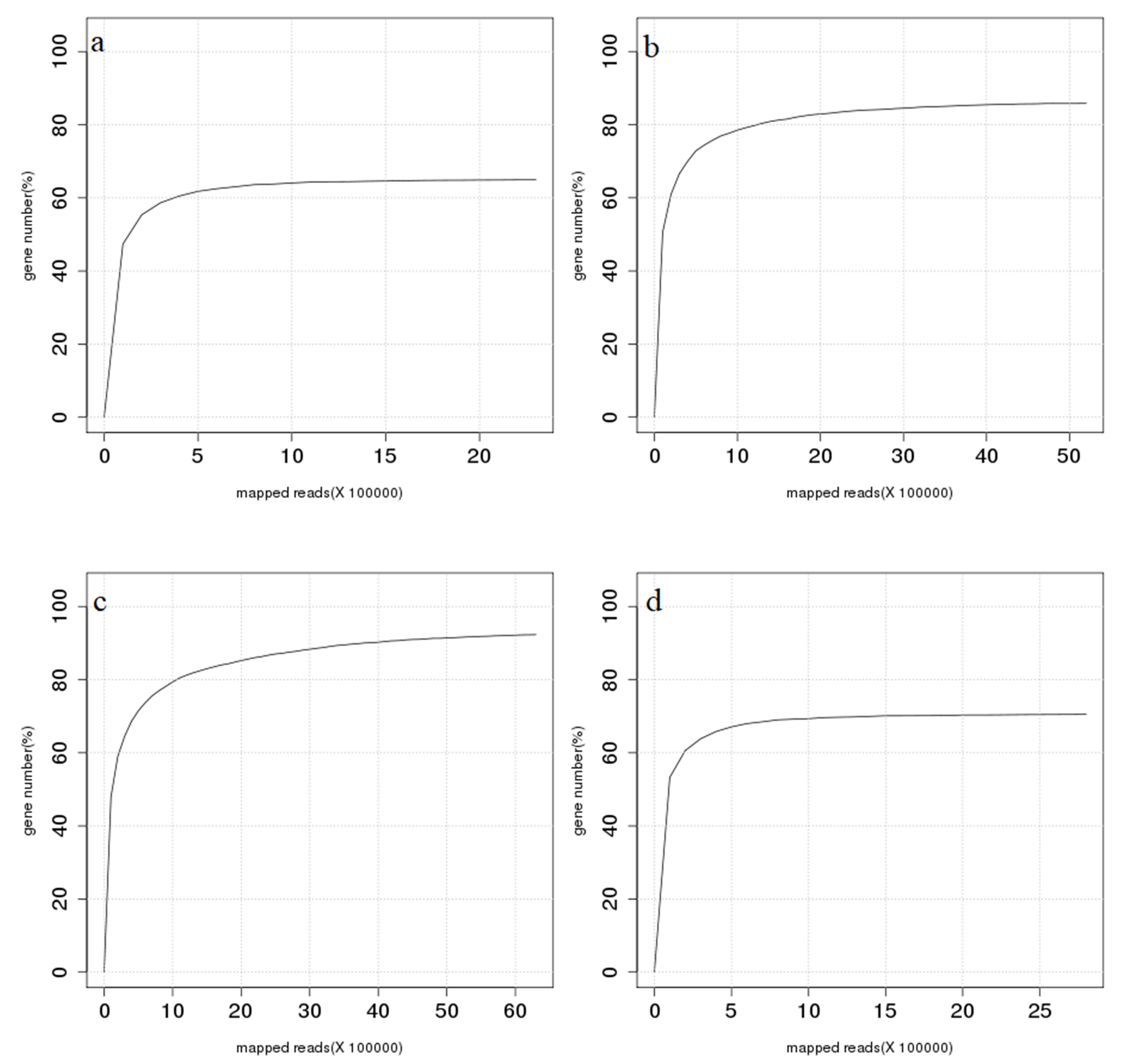


**Fig.S2** Sequencing saturation map of strain R-001.a: E1, b: E2, c:EE2, d: G.


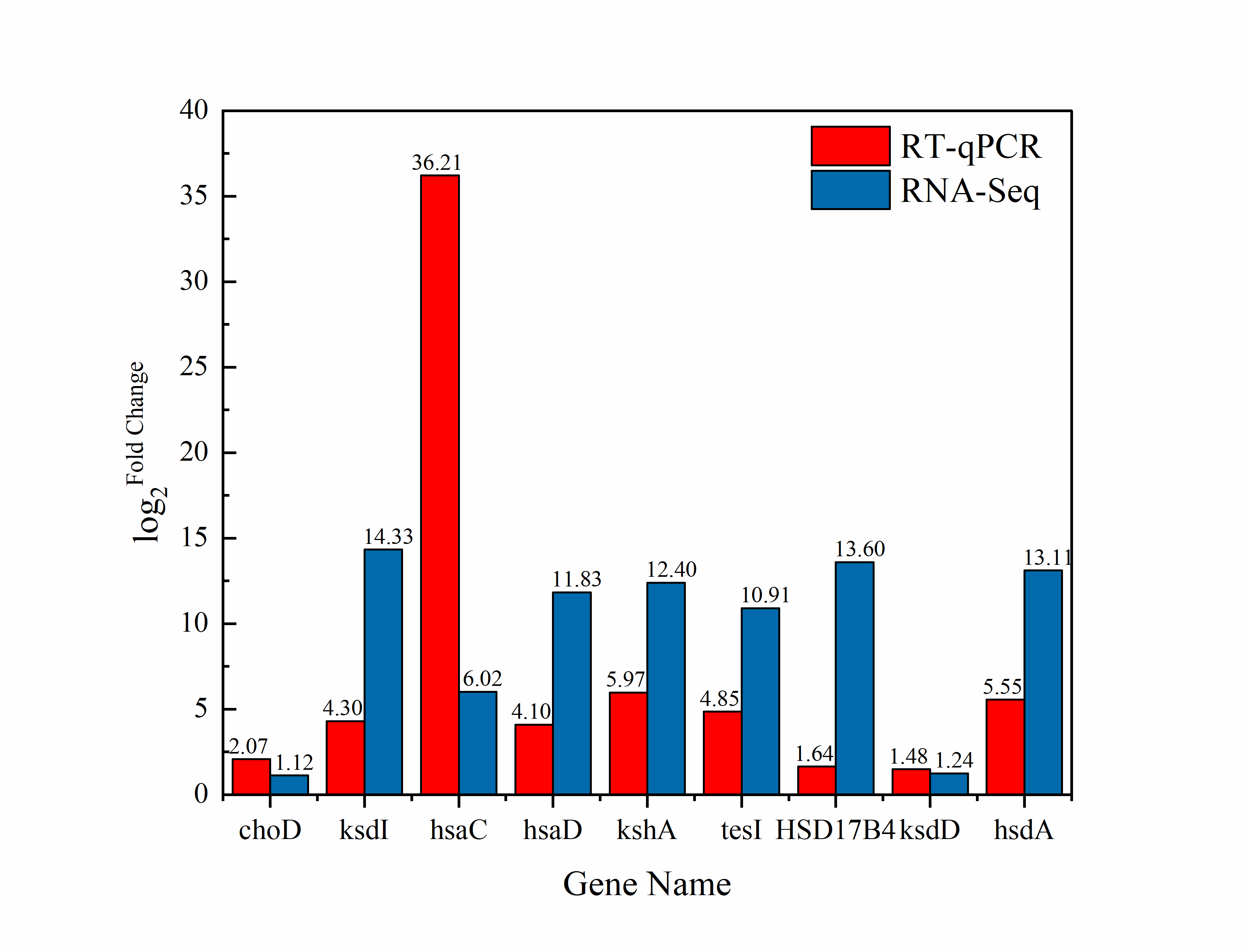


**Fig. S3** Differential expression of genes under estradiol treatment, comparison between RT-qPCR and RNA-seq.


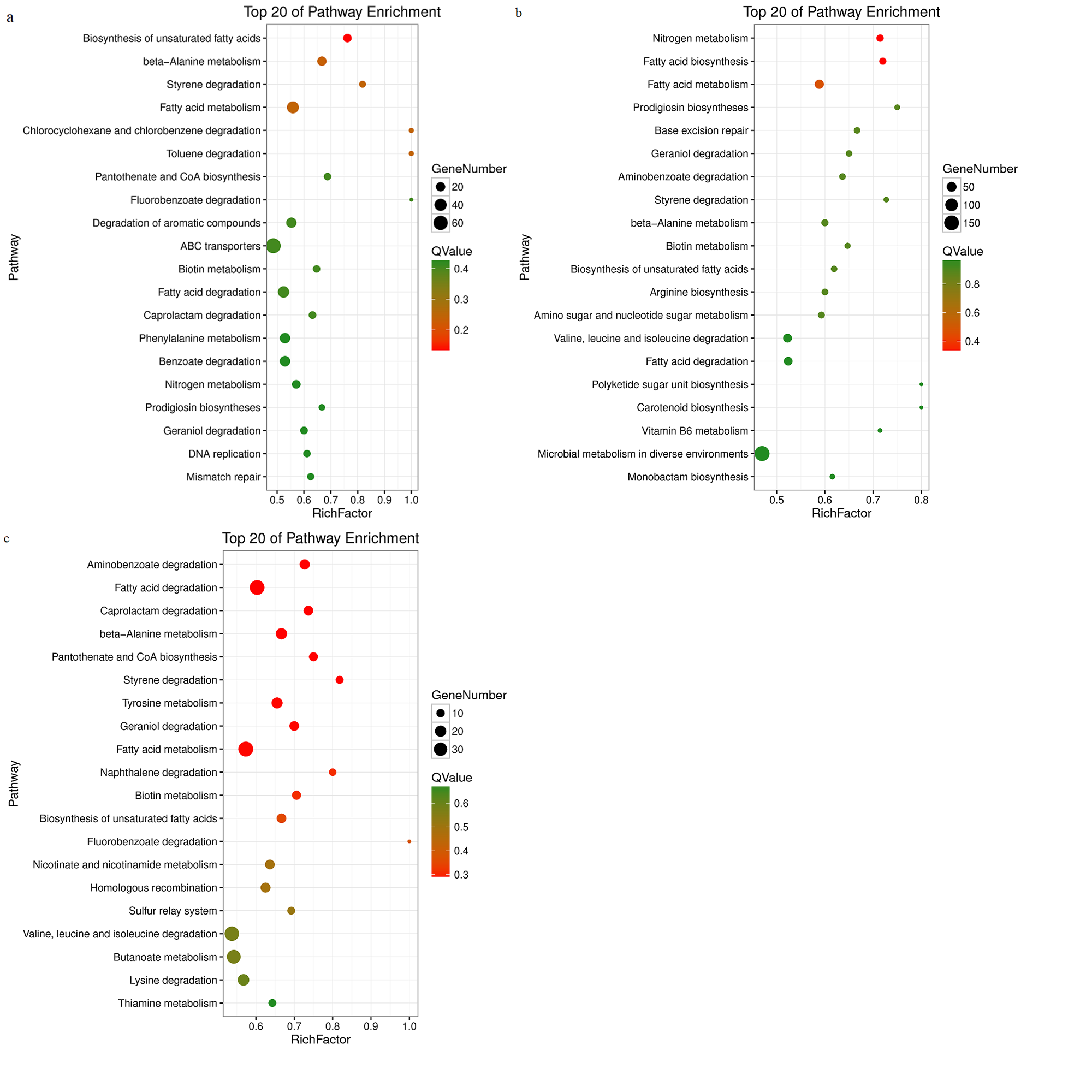


**Fig. S4** Enriched bubblechart of KEGG for R-001 differentially expressed gene under steroid estrogen treatment. a: G-vs-E1, b: G-vs-E2, c: G-vs-EE2. RichFactor refers to the ratio of the number of DEGs to the total number of genes in the pathway. A larger RichFactor indicates a higher degree of enrichment. The closer QValue is to zero, the more significant the enrichment is.


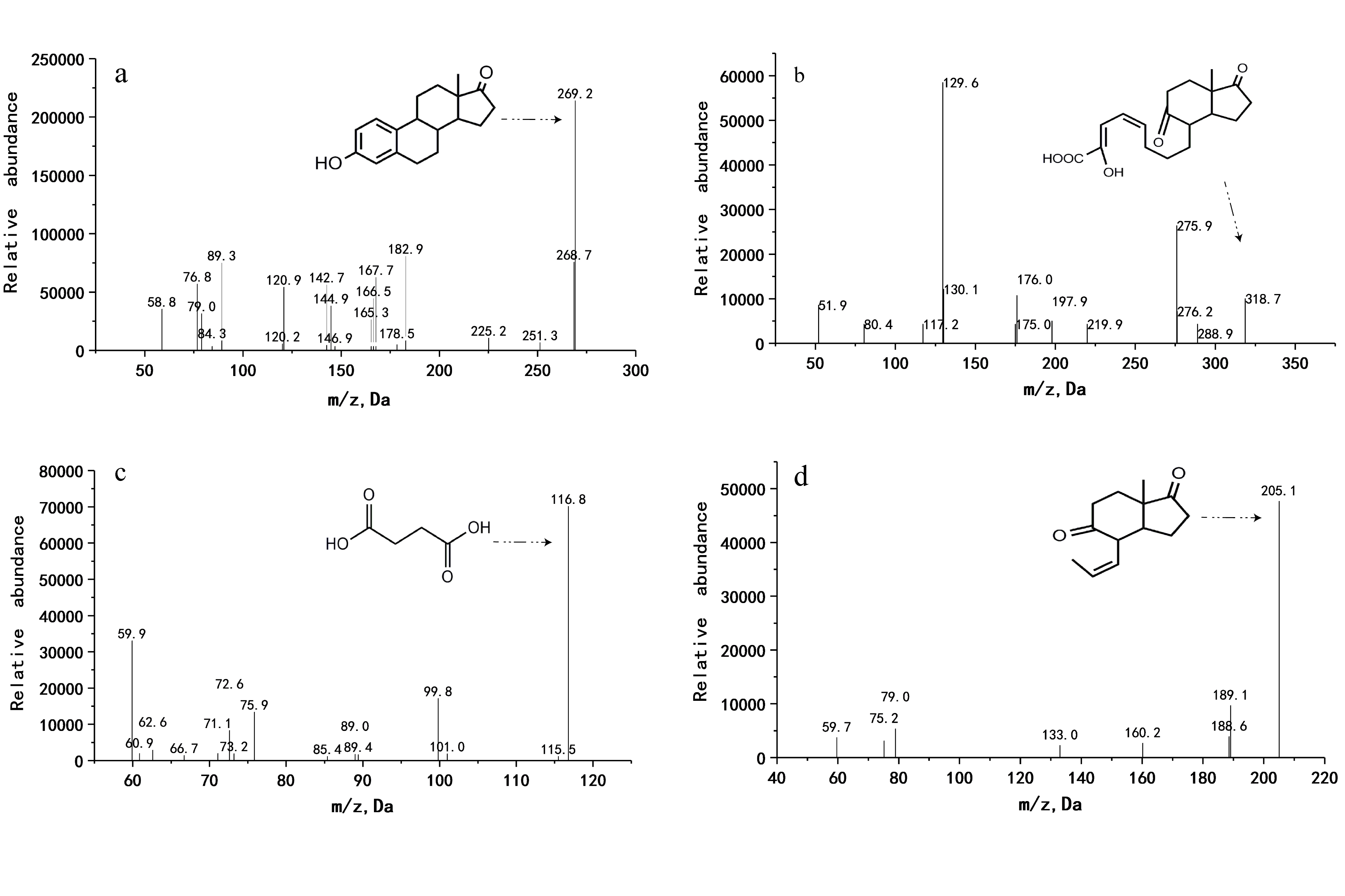


**Fig. S5** Estradiol metabolic pathway map of strain R-001 under E2 treatment. Red border:enzymes encoded by up-regulated genes, green border:enzymes encoded by down-regulated genes, red+green border: enzymes encoded by both up-regulated and down-regulated genes. The main intermediate product in the process of E2 degradation. a:E1, b: 3-hydroxy-4,5-9,10-disecoestrane-1 (10), 2-diene-5,9,17-trione-4-oic acid, c: succinate, d: (Z)-7a-methyl-4-(prop-1-en-1-yl)hexahydro-1H-indole-1,5-(4H) – Diketone.
