# Supplementary material for: The Analysis of Estrogen-Degrading and Functional Metabolism Genes in Rhodococcus equi DSSKP-R-001
Source: Int J Genomics. 2020 Aug 25;2020:9369182. doi: 10.1155/2020/9369182 (PMC7471831; doi:10.1155/2020/9369182)
Supplement: Supplementary 2 — Supplementary tables: Table S1: isolated estrogen-degrading bacteria. Table S2: the primer information in this study. Table S3: qPCR reaction procedure. Table S4: GO function enrichment analysis result. Table S5: DEGs related to metabolism of strain R-001 under different estrogen treatment conditions. [file 9369182.f2.docx]

**Table S1**. Isolated estrogen-degrading bacteria

| Phylogenetic affiliation | Name | Degradation ability  and mechanism | Concentration | Degradation rate/% | Time/h | References |
| --- | --- | --- | --- | --- | --- | --- |
| Alpha-  proteobacteria | *Sphingomonas* sp. ED8 | E1,E2 | E2：0.8 mg/4 mL | 100 | 120 | [24] |
|  |  |  | E1：0.8 mg/4 mL | 90 | 120 |  |
|  | *Sphingomonas* sp. KC8 | E1,E2 | E1：product | 100 | 72 | [15] |
|  |  |  | E2：3 mg/L | 100 | 24 |  |
|  | *Sphingomonas* sp. CYH | E1,E2 | E2：500 μg/L | 100 | 48 | [14] |
|  |  |  | E1：500 μg/L | 100 | 48 |  |
|  | *Novosphingobium tardaugens* ARI-1 | E1, E2, E3 | E3：10 mg/30 mL | 100 | 240 | [10] |
|  | *Phyllobacterium myrsinacearum* BP1 | E1,E2, E3,  co-metabolism EE2 | E1：3.2 mg/L | 99 | 24 | [16] |
|  | *Brevundimonas diminuta* I | E1,E2,EE2 | E2：3.5 mg/L | 100 | 24 | [22] |
|  |  |  | E1：2 mg/L | 95 | 96 |  |
|  |  |  | EE2：3 mg/L | 99 | 360 |  |
| Beta-  proteobacteria | *Ralstonia pickettii* BP2 | E1, E2,E3,  co-metabolism EE2 | E2：2.3 mg/L | 100 | 48 | [16] |
|  | *Ralstonia* sp. *picketii* | E1, E2 |  | 100 | 72 | [12] |
|  | *Achromobacter xylosoxidans* | E1,E2 | E2：1.5 mg/L | 100 | 3 |  |
| Gamma-  proteobacteria | *Phyllobacterium myrsinacearum* BP3 | E1,E2, E3,  co-metabolism EE2 | E1：3.6 mg/L | 99 | 24 | [16] |
|  | *Pseudomonas aeruginosa* TJ1 | E2 | E2：5-15 μg/L | 100 | 2 | [19] |
|  | *Serratia nematodiphila* DH-S01 | E1,E2 | E2：15 mg/L | 93 | 96 | [34] |
|  |  |  | E1 | 93 | 96 |  |
| Firmicutes | *Virgibacillus halotolerans* LF1 | E1,E2 | E2：5 mg/L | 100 | 504 | [31] |
|  |  |  | E1：product | 100 | 768 |  |
|  | *Bacillus flexus* LF3 | E1,E2 | E2：5 mg/L | 98 | 768 |  |
|  | *Bacillus* sp. E2Y1 | E1, E2 | E2：1 mg/L | 100 | 144 | [23] |
|  | *Bacillus* sp. E2Y2 | E2 | E2：1 mg/L | 100 | 96 |  |
|  | *Bacillus* sp. E2Y4 | E1, E2 | E2：1 mg/L | 100 | 144 |  |
|  | *Lysinibacillus sphaericus* DH-B01 |  | E2：30 mg/L | 97 | 96 | [33] |
| Actinobacteria | *Rhodococcus equi* Y50155 | E1, E2, E3, EE2 | E2：100 mg/L | 99 | 24 | [11] |
|  |  |  | E1：100 mg/L | 99 | 24 |  |
|  |  |  | E3：100 mg/L | 72 | 24 |  |
|  |  |  | EE2：100 mg/L | 80 | 24 |  |
|  | *Rhodococcus equi* Y50156 | E1, E2, E3, EE2 | E1：100 mg/L | 99 | 24 |  |
|  |  |  | E2：100 mg/L | 99 | 24 |  |
|  |  |  | E3：100 mg/L | 95 | 24 |  |
|  |  |  | EE2：100 mg/L | 96 | 24 |  |
|  | *Rhodococcus zopfii* Y50158 | E1, E2, E3, EE2 | E1：100 mg/L | 100 | 24 |  |
|  |  |  | E2：100 mg/L | 100 | 24 |  |
|  |  |  | E3：100 mg/L | 100 | 24 |  |
|  |  |  | EE2：100 mg/L | 100 | 24 |  |
|  | *Rhodococcus rubber* KC4 | E2 | E2：3 mg/L | 99 | 24 | [15] |
|  | *Rhodococcus equi* DSSKP-R-001 | E1，E2，E3，EE2 | E1：30 mg/L | 100 | 96 | This study |
|  |  |  | E2：30 mg/L | 100 | 96 |  |
|  |  |  | EE2：30 mg/L | 90 | 72 |  |

**Table.S2** the primers information in this study.

| **Gene** | **Forward sequence (5'-3')** | **Reverse sequence (5'-3')** | **PCR Products(bp)** |
| --- | --- | --- | --- |
| recA | GGACATGCTGATTCGTTCCG | TGGTGCCCGAGTTGTTGAG | 176 |
| choD | GCTGAGGGCAGTGGGAGT | GCGAGTTGTCGTCGGTCTG | 119 |
| ksdI | AACACCGTCGCCTCCTACA | CGTCTGGTCCATCGCCTC | 168 |
| hsaC | GGCAGGAATGGGACGAAT | GCGATTGTCAGCCTTGAAGTA | 101 |
| hsaD | CGACGAGATCCCGCACAA | CACTCGCCGAGGATGTGC | 123 |
| kshA | CGTTCGGAAGCATCCTATTT | AGTTGTCGGTCACGGGGTA | 118 |
| tesI | GCAGGCGGCACTGTTCAA | CTCGGCGAAGCGGTTGTA | 199 |
| HSD17B4 | GACGTGGAGCGACTGAAGC | CGTAGGCGTAGATGGTGGTG | 121 |
| ksdD | CTGGTACAAGGCGGGCATC | CCGTAGTAGCGGTCGTAGGC | 174 |
| hsdA | CGATCCGCTCACCATCAA | GACCGACGAGATCACCACC | 121 |

**Table.S3** qPCR reaction procedure.

| Items | Temperature | Time | Cycle number |
| --- | --- | --- | --- |
| Initial denaturation | 95℃ | 5min | 1 |
| Denaturation | 95℃ | 10sec | 40 |
| Annealing and extension | 60℃ | 30sec |  |
| Melting curve collection | 95℃ | 15sec | 1 |
|  | 60℃ | 60sec |  |
|  | 95℃ | 30sec |  |
|  | 95℃ | 15sec |  |

**Table.S4** GO function enrichment analysis result.

| Estrogen |  | GO ID | Description | Gene (Ratio) | Bgene (Ratio) | Gene Number  (up/down) | Gene ID (up) | Gene ID (down) | p-value |
| --- | --- | --- | --- | --- | --- | --- | --- | --- | --- |
| E1 | Cellular Component | GO:0005623 | cell | 3 (100%) | 16 (80%) | 1/2 | C7H75_RS18270 | C7H75_RS10100; C7H75_RS18640 | 0.491 |
|  |  | GO:0044464 | cell part | 3 (100%) | 16 (80%) | 1/2 | C7H75_RS18270 | C7H75_RS10100; C7H75_RS18640 | 0.491 |
|  |  | GO:0032991 | macromolecular complex | 1 (33.33%) | 9 (45%) | 0/1 |  | C7H75_RS18640 | 0.855 |
|  | Molecular Function | GO:0003824 | catalytic activity | 6 (75%) | 24 (75%) | 1/5 | C7H75_RS12705 | C7H75_RS03905; C7H75_RS10100;  C7H75_RS14070; C7H75_RS14550;  C7H75_RS19660 | 0.691 |
|  |  | GO:0005488 | binding | 3 (37.5%) | 19 (59.38%) | 1/2 | C7H75_RS13185 | C7H75_RS10100; C7H75_RS25170 | 0.969 |
|  | Biological Process | GO:0051179 | localization | 1 (10%) | 3 (8.33%) | 0/1 |  | C7H75_RS04495 | 0.636 |
|  |  | GO:0071840 | cellular component organization or biogenesis | 1(10%) | 4 (11.11%) | 1/0 | C7H75_RS13185 |  | 0.746 |
|  |  | GO:0044699 | Single-organism process | 4 (40%) | 18 (50%) | 0/4 |  | C7H75_RS03905; C7H75_RS10100;  C7H75_RS14550; C7H75_RS19660 | 0.868 |
|  |  | GO:0009987 | cellular process | 4(40%) | 19 (52.78%) | 1/3 | C7H75_RS18270 | C7H75_RS02100; C7H75_RS10100;  C7H75_RS19660 | 0.908 |
|  |  | GO:0008152 | metabolic process | 8 (80%) | 32 (88.89%) | 1/7 | C7H75_RS18270 | C7H75_RS02100; C7H75_RS03905; C7H75_RS10100;  C7H75_RS14070;  C7H75_RS14550; C7H75_RS18640;  C7H75_RS19660 | 0.943 |
| E2 | Cellular Component | GO:0016020 | membrane | 1 (20%) | 4 (20%) | 1/0 | C7H75_RS24350 |  | 0.718 |
|  |  | GO:0005623 | cell | 4(80%) | 16 (80%) | 2/2 | C7H75_RS18270; C7H75_RS18640 | C7H75_RS18615; C7H75_RS20250 | 0.751 |
|  |  | GO:0044464 | cell part | 4 (80%) | 16 (80%) | 2/2 | C7H75_RS18270; C7H75_RS18640 | C7H75_RS18615; C7H75_RS20250 | 0.751 |
|  |  | GO:0032991 | macromolecular complex | 2 (40%) | 9 (45%) | 1/1 | C7H75_RS18640 | C7H75_RS18615 | 0.779 |
|  | Molecular Function | GO:0003824 | catalytic activity | 12 (80%) | 24 (75%) | 4/8 | C7H75_RS06570; C7H75_RS11780; C7H75_RS12080; C7H75_RS22600 | C7H75_RS03905; C7H75_RS08580;  C7H75_RS14070; C7H75_RS14550;  C7H75_RS17865; C7H75_RS18465;  C7H75_RS20250; C7H75_RS25200 | 0.421 |
|  |  | GO:0005198 | structural molecule activity | 1 (6.67%) | 4 (12.5%) | 0/1 |  | C7H75_RS18615 | 0.934 |
|  |  | GO:0005488 | binding | 7 (46.67%) | 19 (59.38%) | 1/6 | C7H75_RS11780 | C7H75_RS06765; C7H75_RS17865;  C7H75_RS18465; C7H75_RS18615;  C7H75_RS25170; C7H75_RS25200 | 0.959 |
|  | Biological Process | GO:0008152 | metabolic process | 14 (93.33%) | 32 (88.89%) | 5/9 | C7H75_RS06570; C7H75_RS11780; C7H75_RS18270; C7H75_RS18640; C7H75_RS22600 | C7H75_RS02100; C7H75_RS03905;  C7H75_RS08580; C7H75_RS14070;  C7H75_RS14550; C7H75_RS17865;  C7H75_RS18615; C7H75_RS20250;  C7H75_RS25200 | 0.440 |
|  |  | GO:0044699 | single-organism process | 8 (53.33%) | 18 (50%) | 3/5 | C7H75_RS06570; C7H75_RS11780; C7H75_RS22600 | C7H75_RS03905; C7H75_RS08580;  C7H75_RS14550; C7H75_RS17865;  C7H75_RS20250 | 0.5 |
|  |  | GO:0051179 | localization | 1 (6.67%) | 3 (8.33%) | 1/0 | C7H75_RS12080 |  | 0.814 |
|  |  | GO:0009987 | cellular process | 7 (46.67%) | 19 (52.78%) | 3/4 | C7H75_RS06570; C7H75_RS11780; C7H75_RS18270 | C7H75_RS02100; C7H75_RS17865;  C7H75_RS20250; C7H75_RS25200 | 0.831 |
|  |  | GO:0071840 | cellular component organization or biogenesis | 1 (6.67%) | 4 (11.11%) | 0/1 |  | C7H75_RS25200 | 0.898 |
| EE2 | Cellular Component | GO:0005623 | cell | 5 (83.33%) | 16 (80%) | 3/2 | C7H75_RS17040; C7H75_RS18270; C7H75_RS18640 | C7H75_RS10100; C7H75_RS18615 | 0.657 |
|  |  | GO:0044464 | cell part | 5 (83.33%) | 16 (80%) | 3/2 | C7H75_RS17040; C7H75_RS18270; C7H75_RS18640 | C7H75_RS10100; C7H75_RS18615 | 0.657 |
|  |  | GO:0016020 | membrane | 1 (16.67%) | 4 (20%) | 1/0 | C7H75_RS24350 |  | 0.793 |
|  |  | GO:0032991 | macromolecular complex | 2(33.33%) | 9 (45%) | 1/1 | C7H75_RS18640 | C7H75_RS18615 | 0.881 |
|  | Molecular Function | GO:0003824 | catalytic activity | 11 (84.62%) | 24 (75%) | 4/7 | C7H75_RS06570; C7H75_RS17865; C7H75_RS19660; C7H75_RS22600 | C7H75_RS03905; C7H75_RS08580;  C7H75_RS09955; C7H75_RS10100;  C7H75_RS14070; C7H75_RS14550;  C7H75_RS18465 | 0.271 |
|  |  | GO:0005198 | structural molecule activity | 1 (7.69%) | 4 (12.5%) | 0/1 |  | C7H75_RS18615 | 0.892 |
|  |  | GO:0005488 | binding | 5 (38.46%) | 19 (59.38%) | 2/3 | C7H75_RS17040; C7H75_RS17865 | C7H75_RS10100; C7H75_RS18465;  C7H75_RS18615 | 0.991 |
|  | Biological Process | GO:0008152 | metabolic process | 14 (100%) | 32 (88.89%) | 7/7 | C7H75_RS06570; C7H75_RS17040; C7H75_RS17865; C7H75_RS18270; C7H75_RS18640; C7H75_RS19660; C7H75_RS22600 | C7H75_RS03905; C7H75_RS08580;  C7H75_RS09955; C7H75_RS10100;  C7H75_RS14070; C7H75_RS14550;  C7H75_RS18615 | 0.124 |
|  |  | GO:0044699 | single-organism process | 9 (64.29%) | 18  (50%) | 5/4 | C7H75_RS06570; C7H75_RS17040; C7H75_RS17865; C7H75_RS19660; C7H75_RS22600 | C7H75_RS03905; C7H75_RS08580;  C7H75_RS10100; C7H75_RS14550 | 0.153 |
|  |  | GO:0009987 | cellular process | 7  (50%) | 19 (52.78%) | 5/2 | C7H75_RS06570; C7H75_RS17040; C7H75_RS17865; C7H75_RS18270; C7H75_RS19660 | C7H75_RS09955; C7H75_RS10100 | 0.728 |
|  |  | GO:0071840 | cellular component organization or biogenesis | 1  (7.14%) | 4 (11.11%) | 1/0 | C7H75_RS17040 |  | 0.876 |

**Table.S5** DEGs related to metabolism of strain R-001 under different estrogen treatment conditions.

| Gene ID | Gene Name | Description | KEGG_B_class | Pathway | log_2_(FC) | | |
| --- | --- | --- | --- | --- | --- | --- | --- |
|  |  |  |  |  | E1 | E2 | EE2 |
| C7H75_RS17810 | ALDH7A1 | aldehyde dehydrogenase family protein | Amino acid metabolism | Valine, leucine and isoleucine degradation | 1.57 |  |  |
| C7H75_RS00395 | ACAD11 | acyl-CoA dehydrogenase | Amino acid metabolism | Valine, leucine and isoleucine degradation | 12.5 | 12.22 | 11.03 |
| C7H75_RS17090 | Acads | acyl-CoA dehydrogenase | Amino acid metabolism | Valine, leucine and isoleucine degradation | 2.38 | 1.58 | 1.63 |
| C7H75_RS03725 | fadA5 | acetyl-CoA acetyltransferase | Amino acid metabolism | Valine, leucine and isoleucine degradation | 2.16 |  | 2.07 |
| C7H75_RS21150 | fadI | 3-ketoacyl-CoA thiolase | Amino acid metabolism | Valine, leucine and isoleucine degradation | 2.86 | 3.27 | 2.88 |
| C7H75_RS08570 | yngG | hydroxymethylglutaryl-CoA lyase | Amino acid metabolism | Valine, leucine and isoleucine degradation | 1.83 | 1.31 | 1.34 |
| C7H75_RS18765 | accA1 | acetyl/propionyl-CoA carboxylase subunit alpha | Amino acid metabolism | Valine, leucine and isoleucine degradation | 1.04 | 1.26 |  |
| C7H75_RS03700 | Msed_2001 | enoyl-CoA hydratase/isomerase | Amino acid metabolism | Valine, leucine and isoleucine degradation | 3.22 |  | 2.31 |
| C7H75_RS02660 | echA14 | enoyl-CoA hydratase | Amino acid metabolism | Valine, leucine and isoleucine degradation | 13.53 |  | 11.6 |
| C7H75_RS20550 | ilvE | branched chain amino acid aminotransferase | Amino acid metabolism | Valine, leucine and isoleucine degradation | 1.28 |  | 1.51 |
| C7H75_RS25410 | pcaF | acetyl-CoA C-acetyltransferase | Amino acid metabolism | Valine, leucine and isoleucine degradation |  | 14.18 | 22.02 |
| C7H75_RS07675 | patD | Zn-dependent alcohol dehydrogenase | Amino acid metabolism | Tyrosine metabolism |  | 16.14 | 13.03 |
| C7H75_RS23550 | sad | NAD-dependent succinate-semialdehyde dehydrogenase | Amino acid metabolism | Tyrosine metabolism |  | 12.12 | 10.75 |
| C7H75_RS22015 | fadB2 | 3-hydroxybutyryl-CoA dehydrogenase | Amino acid metabolism | Phenylalanine metabolism | 2.08 | 1.8 | 2.25 |
| C7H75_RS15410 | gatA | amidase | Amino acid metabolism | Phenylalanine metabolism | 11.83 | 11.23 | 11.87 |
| C7H75_RS02660 | echA14 | enoyl-CoA hydratase | Amino acid metabolism | Phenylalanine metabolism | 13.53 |  | 11.6 |
| C7H75_RS03700 | Msed_2001 | enoyl-CoA hydratase/isomerase | Amino acid metabolism | Phenylalanine metabolism | 3.22 |  | 2.31 |
| C7H75_RS03870 | Msed_2001 | enoyl-CoA hydratase/isomerase | Amino acid metabolism | Phenylalanine metabolism |  | 13.67 | 12.9 |
| C7H75_RS18285 | bphA1 | aromatic ring-opening dioxygenase | Amino acid metabolism | Phenylalanine metabolism | 12.34 |  | 11.46 |
| C7H75_RS18310 | bphB | 3-(cis-5,6-dihydroxycyclohexa-1,3-dien-1-yl) propanoate dehydrogenase | Amino acid metabolism | Phenylalanine metabolism | 14.77 | 13.79 | 13.36 |
| C7H75_RS22860 | mhpA2 | aromatic ring hydroxylase | Amino acid metabolism | Phenylalanine metabolism | 1.6 |  | 1.14 |
| C7H75_RS25420 | Swol_1936 | enoyl-CoA hydratase | Amino acid metabolism | Phenylalanine metabolism |  | 5.21 | 13.03 |
| C7H75_RS02550 | amnD | endoribonuclease | Amino acid metabolism | Tryptophan metabolism | 15.47 | 13.79 | 15.15 |
| C7H75_RS03725 | fadA5 | acetyl-CoA acetyltransferase | Amino acid metabolism | Tryptophan metabolism | 2.16 |  | 2.07 |
| C7H75_RS21150 | fadI | 3-ketoacyl-CoA thiolase | Amino acid metabolism | Tryptophan metabolism | 2.86 | 3.27 | 2.88 |
| C7H75_RS15410 | gatA | amidase | Amino acid metabolism | Tryptophan metabolism | 11.83 | 11.23 | 11.87 |
| C7H75_RS03700 | Msed_2001 | enoyl-CoA hydratase/isomerase | Amino acid metabolism | Tryptophan metabolism | 3.22 |  | 2.31 |
| C7H75_RS11510 | mdh | L-malate dehydrogenase | Amino acid metabolism | Cysteine and methionine metabolism | 1.95 |  | 1.41 |
| C7H75_RS19880 | metE | 5-methyltetrahydropteroyltriglutamate--homocysteine S-methyltransferase | Amino acid metabolism | Cysteine and methionine metabolism | 1.51 | 1.44 | 1.59 |
| C7H75_RS24755 | dsaVM | Site-specific DNA methylase | Amino acid metabolism | Cysteine and methionine metabolism | 15.5 | 14.26 | 14.26 |
| C7H75_RS24785 | apaLIM | DNA cytosine methyltransferase | Amino acid metabolism | Cysteine and methionine metabolism | 13.12 | 12.94 | 13.71 |
| C7H75_RS21270 | cysE | serine O-acetyltransferase | Amino acid metabolism | Cysteine and methionine metabolism | 1.86 | 1.68 | 1.86 |
| C7H75_RS20550 | ilvE | branched chain amino acid aminotransferase | Amino acid metabolism | Cysteine and methionine metabolism | 1.28 |  | 1.51 |
| C7H75_RS04510 | ttuD | thiosulfate sulfurtransferase | Amino acid metabolism | Cysteine and methionine metabolism | 1.59 |  | 2.25 |
| C7H75_RS21275 | cysK1 | cysteine synthase CysK/M | Amino acid metabolism | Cysteine and methionine metabolism |  | 1.48 | 1.78 |
| C7H75_RS07150 | egtA | ergothioneine biosynthesis glutamate--cysteine ligase EgtA | Amino acid metabolism | Cysteine and methionine metabolism | 1.82 | 2.84 |  |
| C7H75_RS15675 | ectB | diaminobutyrate-2-oxoglutarate aminotransferase EctB | Amino acid metabolism | Glycine, serine and threonine metabolism |  | 13.29 | 11.96 |
| C7H75_RS23550 | sad | NAD-dependent succinate-semialdehyde dehydrogenase | Amino acid metabolism | Lysine degradation |  | 12.12 | 10.75 |
| C7H75_RS03725 | fadA5 | acetyl-CoA acetyltransferase | Amino acid metabolism | Lysine degradation | 2.16 |  | 2.07 |
| C7H75_RS21150 | fadI | 3-ketoacyl-CoA thiolase | Amino acid metabolism | Lysine degradation | 2.86 | 3.27 | 2.88 |
| C7H75_RS02660 | echA14 | enoyl-CoA hydratase | Amino acid metabolism | Lysine degradation | 13.53 |  | 11.6 |
| C7H75_RS03700 | Msed_2001 | enoyl-CoA hydratase/isomerase | Amino acid metabolism | Lysine degradation | 3.22 |  | 2.31 |
| C7H75_RS03870 | Msed_2001 | enoyl-CoA hydratase/isomerase | Amino acid metabolism | Lysine degradation |  | 13.67 | 12.9 |
| C7H75_RS04390 |  | dihydrodipicolinate reductase | Amino acid metabolism | Lysine degradation | 13.16 | 11.39 |  |
| C7H75_RS17045 | hisN | histidinol-phosphatase | Amino acid metabolism | Histidine metabolism | 1.89 | 2.35 | 1.87 |
| C7H75_RS23550 | sad | NAD-dependent succinate-semialdehyde dehydrogenase | Amino acid metabolism | Alanine, aspartate and glutamate metabolism |  | 12.12 | 10.75 |
| C7H75_RS00225 | gltB | glutamate synthase subunit alpha | Amino acid metabolism | Alanine, aspartate and glutamate metabolism | 2.02 | 1.74 | 2.5 |
| C7H75_RS00230 | gltD | glutamate synthase small subunit GltB1 | Amino acid metabolism | Alanine, aspartate and glutamate metabolism | 1.27 | 2.4 | 1.73 |
| C7H75_RS20480 | glnT | type III glutamate--ammonia ligase | Amino acid metabolism | Alanine, aspartate and glutamate metabolism |  | 11.71 | 12.09 |
| C7H75_RS15025 | glnA | glutamate-ammonia ligase GlnA1 | Amino acid metabolism | Alanine, aspartate and glutamate metabolism | 2.07 |  | 1.29 |
| C7H75_RS23510 | glnA | glutamine synthetase | Amino acid metabolism | Alanine, aspartate and glutamate metabolism | 13.83 | 12.76 | 11.42 |
| C7H75_RS15700 | proB | glutamate 5-kinase ProB | Amino acid metabolism | Arginine and proline metabolism |  | 2.01 | 1.05 |
| C7H75_RS14985 | pepA | leucyl aminopeptidase | Amino acid metabolism | Arginine and proline metabolism | 1.43 | 2.01 | 1.03 |
| C7H75_RS15410 | gatA | amidase | Amino acid metabolism | Arginine and proline metabolism | 11.83 | 11.23 | 11.87 |
| C7H75_RS07675 | patD | Zn-dependent alcohol dehydrogenase | Carbohydrate metabolism | Glycolysis / Gluconeogenesis |  | 16.14 | 13.03 |
| C7H75_RS02065 | pyk | pyruvate kinase | Carbohydrate metabolism | Glycolysis / Gluconeogenesis | 13.79 |  | 11.26 |
| C7H75_RS19795 | deoC | deoxyribose-phosphate aldolase | —— | Pentose phosphate pathway |  | 13.45 | 12.31 |
| C7H75_RS02075 | fabG | 3-oxoacyl-ACP reductase | lipid metabolism | Fatty acid metabolism | 13.37 |  | 11.73 |
| C7H75_RS02080 | fabG | short-chain dehydrogenase/reductase | lipid metabolism | Fatty acid metabolism | 12.7 | 13.18 | 12.45 |
| C7H75_RS02695 | fabG | short-chain dehydrogenase/reductase | lipid metabolism | Fatty acid metabolism | 13.58 | 12.75 |  |
| C7H75_RS08635 | fabG | 3-oxoacyl-ACP reductase | lipid metabolism | Fatty acid metabolism | 14.04 | 13.41 | 11.94 |
| C7H75_RS21155 | fabG | short chain dehydrogenase | lipid metabolism | Fatty acid metabolism | 2.99 | 2.88 | 2.83 |
| C7H75_RS22600 | fox-2 | 3-oxoacyl-ACP reductase | lipid metabolism | Fatty acid metabolism |  | 13.31 | 11.55 |
| C7H75_RS22690 | novJ | short chain dehydrogenase | lipid metabolism | Fatty acid metabolism | 13.15 | 14.18 | 11.86 |
| C7H75_RS00395 | ACAD11 | acyl-CoA dehydrogenase | lipid metabolism | Fatty acid metabolism |  | 12.22 | 11.03 |
| C7H75_RS17090 | Acads | acyl-CoA dehydrogenase | lipid metabolism | Fatty acid metabolism | 2.38 | 1.58 | 1.63 |
| C7H75_RS03725 | fadA5 | acetyl-CoA acetyltransferase | lipid metabolism | Fatty acid metabolism | 2.16 |  | 2.07 |
| C7H75_RS21150 | fadI | 3-ketoacyl-CoA thiolase | lipid metabolism | Fatty acid metabolism | 2.86 | 3.27 | 2.88 |
| C7H75_RS08365 | fadA | acetyl-CoA acetyltransferase | lipid metabolism | Fatty acid metabolism | 3.8 |  | 3.91 |
| C7H75_RS02660 | echA14 | enoyl-CoA hydratase | lipid metabolism | Fatty acid metabolism | 13.53 |  | 11.6 |
| C7H75_RS03700 | Msed_2001 | enoyl-CoA hydratase/isomerase | lipid metabolism | Fatty acid metabolism | 3.22 |  | 2.31 |
| C7H75_RS03870 | Msed_2001 | enoyl-CoA hydratase/isomerase | lipid metabolism | Fatty acid metabolism |  | 13.67 | 12.9 |
| C7H75_RS08795 | fadD15 | acyl-CoA ligase | lipid metabolism | Fatty acid metabolism | 1.04 | 1.17 |  |
| C7H75_RS04590 | S-ACP-DES6 | fatty acid desaturase | lipid metabolism | Fatty acid metabolism | 3.78 | 2.15 | 3.14 |
| C7H75_RS18765 | accA1 | acetyl/propionyl-CoA carboxylase subunit alpha | lipid metabolism | Fatty acid metabolism | 1.04 | 1.26 |  |
| C7H75_RS17430 | desA3 | fatty acid desaturase | lipid metabolism | Fatty acid metabolism | 1.23 | 1.64 | 1.49 |
| C7H75_RS11510 | mdh | L-malate dehydrogenase | —— | Pyruvate metabolism | 1.95 |  | 1.41 |
| C7H75_RS07995 | poxB | thiamine pyrophosphate-requiring protein | —— | Pyruvate metabolism |  | 2.16 | 1.79 |
| C7H75_RS03725 | fadA5 | acetyl-CoA acetyltransferase | —— | Pyruvate metabolism | 2.16 |  | 2.07 |
| C7H75_RS21150 | fadI | 3-ketoacyl-CoA thiolase | —— | Pyruvate metabolism | 2.86 | 3.27 | 2.88 |
| C7H75_RS25410 | pcaF | acetyl-CoA C-acetyltransferase | —— | Pyruvate metabolism |  | 14.18 | 22.01 |
| C7H75_RS02065 | pyk | pyruvate kinase | —— | Pyruvate metabolism |  | 11.41 | 11.26 |
| C7H75_RS19890 | icl | isocitrate lyase | —— | Pyruvate metabolism | 1.64 | 1.16 | 1.53 |
| C7H75_RS23385 | cat1 | acetyl-CoA hydrolase | —— | Pyruvate metabolism | 1.27 | 1.63 |  |
| C7H75_RS11510 | mdh | L-malate dehydrogenase | —— | TCA cycle | 1.95 |  | 1.41 |
| C7H75_RS14375 | tfrA | fumarate reductase/succinate dehydrogenase flavoprotein subunit | —— | TCA cycle | 1.39 | 1.96 |  |
| C7H75_RS23385 | cat1 | acetyl-CoA hydrolase | —— | TCA cycle | 1.63 | 1.27 |  |
| C7H75_RS14375 | tfrA | fumarate reductase/succinate dehydrogenase flavoprotein subunit | —— | oxidative phosphorylation | 1.39 | 1.96 |  |
| C7H75_RS10520 | nuoN | NADH-quinone oxidoreductase subunit N | —— | oxidative phosphorylation | 3.41 | 2.71 |  |
